# Cross-sectional analyses of metabolites across biological samples mediating dietary acid load and chronic kidney disease

**DOI:** 10.1016/j.isci.2024.109132

**Published:** 2024-02-05

**Authors:** Ilias Attaye, Beverley Beynon-Cobb, Panayiotis Louca, Ana Nogal, Alessia Visconti, Francesca Tettamanzi, Kari Wong, Gregory Michellotti, Tim D. Spector, Mario Falchi, Jordana T. Bell, Cristina Menni

**Affiliations:** 1 Department of Twin Research, King’s College London, St Thomas' Hospital Campus, London SE1 7EH, UK; 2 Amsterdam Cardiovascular Sciences, Diabetes & Metabolism, Amsterdam, the Netherlands; 3 Department of Nutrition & Dietetics, University Hospitals Coventry & Warwickshire NHS Trust, Coventry CV2 2DX, UK; 4 Metabolon, Research Triangle Park, Morrisville, NC 27560, USA

**Keywords:** Health sciences, Medicine, Medical specialty, Internal medicine, Nephrology, Natural sciences, Biological sciences, Systems biology, Metabolomics

## Abstract

Chronic kidney disease (CKD) is a major public health burden, with dietary acid load (DAL) and gut microbiota playing crucial roles. As DAL can affect the host metabolome, potentially via the gut microbiota, we cross-sectionally investigated the interplay between DAL, host metabolome, gut microbiota, and early-stage CKD (TwinsUK, n = 1,453). DAL was positively associated with CKD stage G1-G2 (Beta (95% confidence interval) = 0.34 (0.007; 0.7), p = 0.046). After adjusting for covariates and multiple testing, we identified 15 serum, 14 urine, 8 stool, and 7 saliva metabolites, primarily lipids and amino acids, associated with both DAL and CKD progression. Of these, 8 serum, 2 urine, and one stool metabolites were found to mediate the DAL-CKD association. Furthermore, the stool metabolite 5-methylhexanoate (i7:0) correlated with 26 gut microbial species. Our findings emphasize the gut microbiota’s therapeutic potential in countering DAL’s impact on CKD through the host metabolome. Interventional and longitudinal studies are needed to establish causality.

## Highlights

• Multi-biological sample metabolites associate with dietary acid load and kidney function
• Multiple associated metabolites mediated between DAL and mild kidney function decline
• The stool metabolite, 5-methylhexanoate(i7:0), correlated with several gut microbial species

## Graphical abstract

**Figure:**
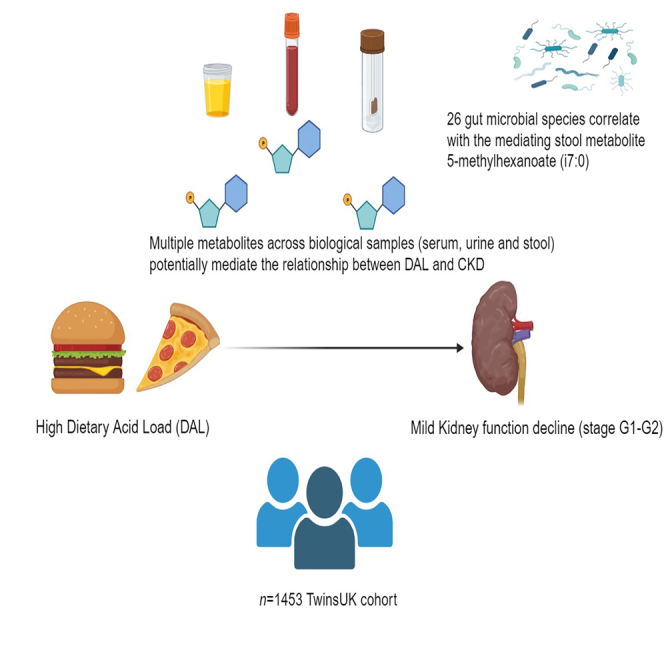

## Introduction

Chronic kidney disease (CKD) is a major cause of morbidity and mortality, with incident rates increasing globally.^1^ Multiple modifiable risk factors for CKD development have been identified, including diet and gut microbiota composition and function.^2^^,^^3^ Recently, there has been an evidence-based shift toward manipulating dietary acid load (DAL), when managing CKD progression.^4^ DAL is defined as the difference (in mEq H^+^/day) between endogenously produced acid and base, originating from diet.^4^ Modern Western diets generally contain large amounts of acid-forming protein, processed foods, and limited amounts of base-producing fruits and vegetables, thereby inducing a state of chronic metabolic acidosis.^5^ An almost linear relationship exists between acidosis and poorer clinical outcomes in individuals with CKD, including CKD progression and mortality.^6^

The mechanisms by which DAL contributes to CKD are complex and poorly understood but have been reported to lead to vascular dysfunction and increased peripheral insulin resistance.^7^^,^^8^^,^^9^

Indeed, a systematic review and meta-analysis of 31 observational studies reported that a higher DAL is associated with increased systolic and diastolic blood pressure.^10^ Increased DAL calculated via potential renal acid load (PRAL) methods was also associated with impaired fasting glucose and increased levels of HbA1c in this study.

However, the underlying metabolic pathways resulting in this dysregulation remain unclear. Metabolomics, a high-throughput technology that provides a snapshot of an individual’s metabolic profile at a particular time point, can be used to provide insights into these pathways and has been used successfully to identify novel markers of CKD risk.^11^^,^^12^ Recently, Tariq and colleagues (2022) identified circulating levels of the amino acid N-methylproline to be inversely associated with both DAL and CKD (stage G3-G4), suggesting a protective effect of diet.^13^

Nevertheless, the effect of DAL on the host metabolome of individuals with early-stage CKD remains largely unexplored. Moreover, the role of the gut microbiome in this relationship is unknown. It is paramount to identify molecular pathways and gut microbiota signatures regulating the DAL-CKD association, as this could lead to effective strategies for mitigating kidney function decline.

Therefore, the aims of this large population-based study were to (i) explore the relationship between DAL and early-stage CKD (stage G1-G2), (ii) investigate the biological pathways underlying this association using metabolomics profiling from four biological samples (serum, urine, stool, and saliva) with mediation analyses to estimate effects, and (iii) assess the interaction between DAL and gut microbiota composition in kidney function decline.

## Results

**Figure 1:**
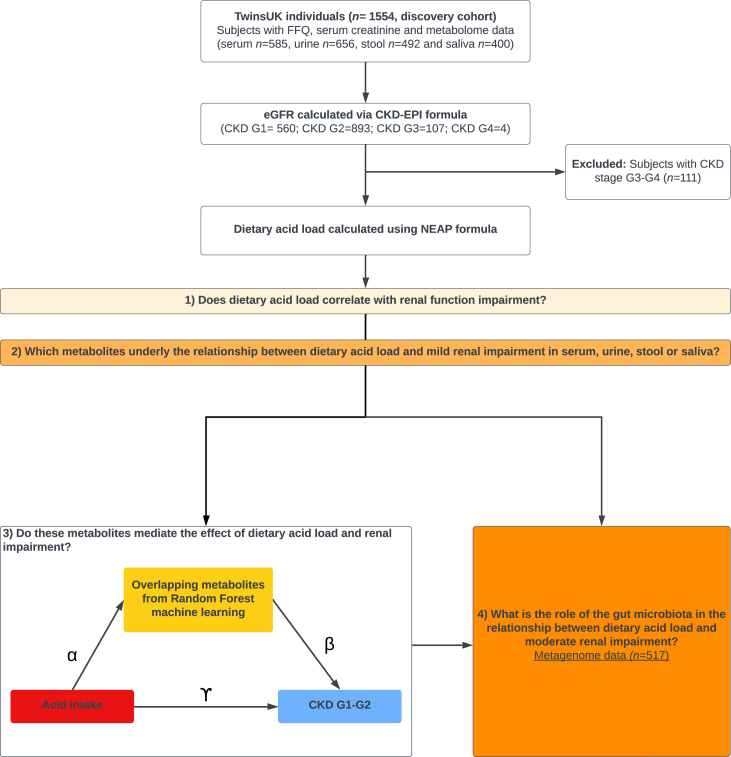
Flow chart of the study design

The descriptive characteristics of the study population are presented in Table 1. This study included 1,453 individuals from TwinsUK with metabolomics profiling available in serum (fasting), urine (spot), stool, and saliva, as well as estimated glomerular filtration rate (eGFR) and dietary data available. Briefly, the predominantly female (89.7%) sample included 560 individuals with CKD stage G1 and 893 CKD stage G2, and on average they were 61.3 (±12) years of age with an average body mass index (BMI) of 25.9 (±4.7) kg/m^2^. A flowchart of the study design is presented in Figure 1.

**Table 1:** Demographic characteristics of the study population overall and by CKD status

	Overall *(*n = 1453)		CKD G1 *(*n = 560)		CKD G2 *(*n = 893)	
	*n*	%	*n*	%	*n*	%
Females/males	1,303/150		503/57		800/93	
CKD stage G1	560	38.5	560	100		
CKD stage G2	893	61.5			893	100

	Mean	SD	Mean	SD	Mean	SD
Age (years)	61.3	12.0	55.8	12.7	64.7	10.2
Energy (kcal/day)	1,864	556.2	1,842	554.7	1,878	557.0
BMI (kg/m2)	25.9	4.74	24.93	4.49	26.5	4.79
Dietary acid (mEq H^+^/day)	51.7	9.5	51.8	9.8	51.7	9.3

### High DAL is associated with renal function decline

Linear mixed models adjusting for age, sex, BMI, and family relatedness identified a positive association between DAL and CKD (β = 0.337 (confidence interval [CI] 0.007; 0.7), p = 0.046). Results were consistent when exploring eGFR as a continuous variable (β = −1.45 (CI −2.89; −0.0047), p = 0.049).

### DAL and renal function exhibit a coordinated signature on the serum, urine, and stool metabolome

To explore the serum, urine, stool, and saliva metabolite profiles of individuals in CKD stage G1 and G2 in relation to their DAL, we performed principal-component analysis and Permutational multivariate analysis of variance (PERMANOVA). We found that the metabolome profile of all biological samples, except for saliva, clustered differently according to their DAL on a global scale (p < 0.05; Figure S1).

**Figure 2:**
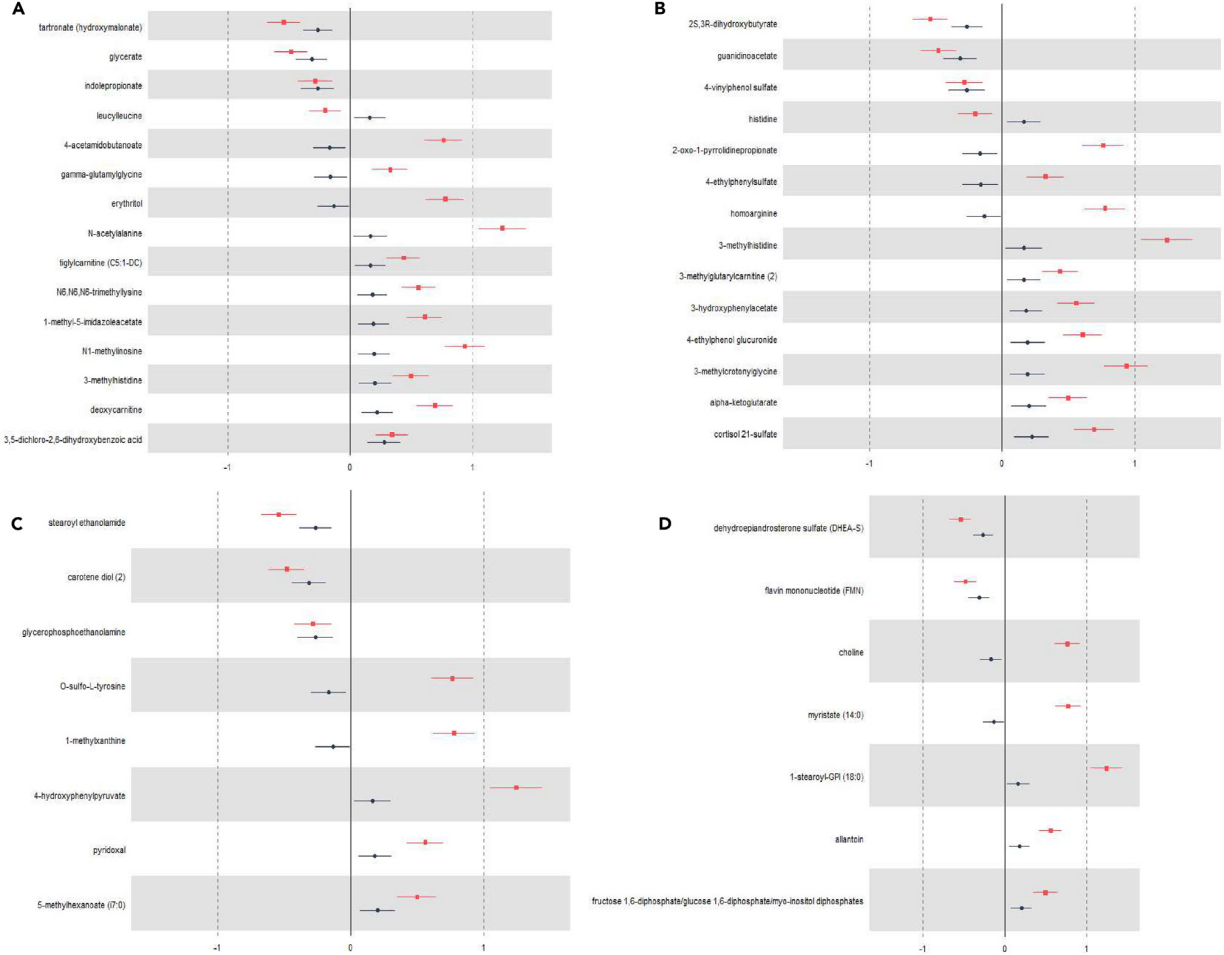
Metabolites associated with CKD and acid intake Metabolites identified through random forest machine learning that pass multivariable regression analysis (p < 0.05, FDR corrected [Benjamini & Hochberg]) and are associated with CKD (red) and acid intake (blue) in (A) serum, (B) urine, (C) stool, and (D) saliva. All analyses were corrected for age, sex, and BMI.

We then performed random forest (RF) models on the residuals-adjusted metabolites and identified 41 metabolites in serum that overlap between CKD and DAL, of which 15 passed FDR correction, 41 in urine, of which 14 passed FDR correction, 27 in stool, of which 8 passed FDR correction, and 22 in saliva, of which 7 passed FDR correction (Figure 2; Table S6).

### Effect of DAL on renal function is mediated through serum, urine, and stool metabolites

**Figure 3:**
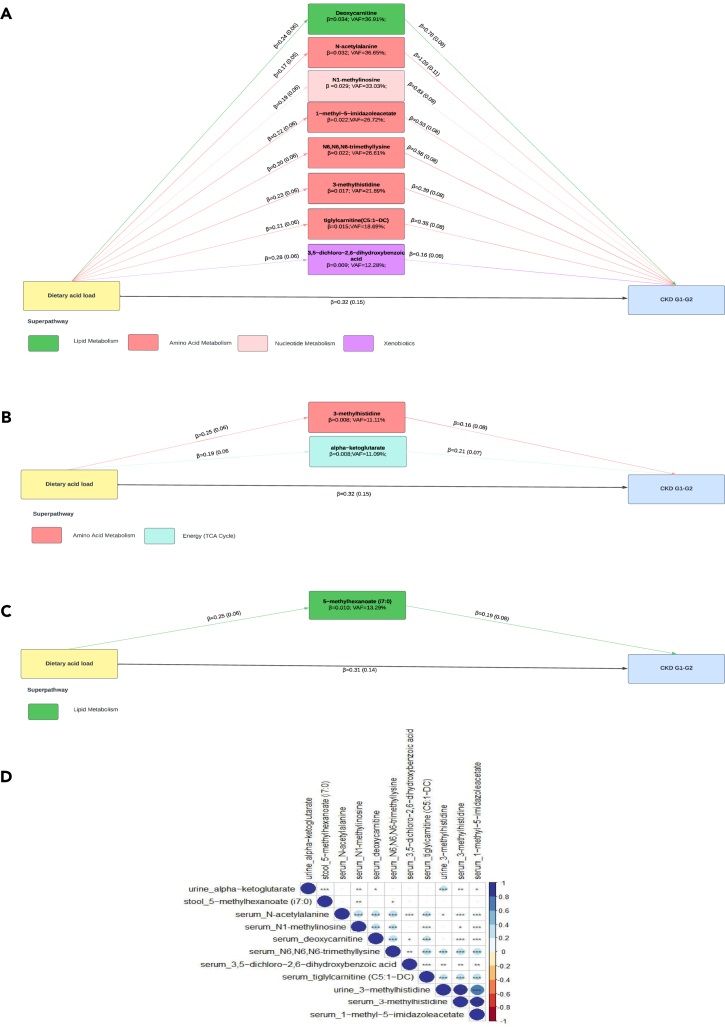
Mediation analysis between DAL and CKD stage G1-G2 Mediation analysis of the association between DAL and CKD stage G1-G2, using metabolite of interest as potential mediator in (A) serum,(B) urine, and (C) stool. Path coefficients are illustrated beside each path and variance accounted for (VAF) score is denoted below the mediator. All associations are statistically significant (p < 0.05). (D) Spearman correlation between mediating metabolites across biological samples (∗p < 0.05; ∗∗p < 0.01 and ∗∗∗p < 0.001). All analyses are corrected for age, sex, and BMI. Abbreviations: DAL, dietary acid load; CKD, chronic kidney disease; VAF, variance accounted for.

We next tested if the overlapping metabolites associated with both DAL and early-stage CKD potentially mediated the association between DAL and CKD stage G1-G2 progression through mediation analysis, correcting for confounding factors such as age, sex, and BMI. We tested both the DAL→metabolite of interest→CKD stage G1-G2 as well as the DAL→CKD stage G1-G2→ metabolite of interest order (Figures 3 and S2).

All 8 positively associated metabolites in serum mediated the effect of DAL and stage G1-G2 progression, and the variance accounted for (VAF) ranged between 12.28% (3,5-dichloro-2,6-dihydrpxybenzoic acid) to 36.91% (deoxycarnitine). Interestingly, 3-methlyhistidine was the only mediating metabolite which was found in both serum (VAF 21.89%) and urine (VAF 11.11% [Figure 3A]).

In urine, only 2/7 positively associated metabolites mediated the effect with VAF ranging from 11.09% (alpha-ketoglutorate) to 11.11% (3-methylhistidine [3-MH]) (Figure 3B). In stool, only 1/3 metabolite, 5-methylhexanoate (i7:0), mediated the association between DAL and CKD stage G1-G2 progression with a VAF of 13.29% (Figure 3C). We did not observe evidence for mediation of the 3 saliva metabolites, which corresponds with the results of the principal-component analysis (Figure S1).

When considering CKD stage G1-G2 as a potential mediator in the relationship between DAL→ metabolite of interest, we found that 7/8 serum metabolites, 1 urine metabolite, and 1 stool metabolite were mediated by CKD stage (Figure S2). The VAF was smaller in this model compared to the model using metabolites of interest as potential mediators (Figure 3). The VAF had a range from 8.38% (tiglylcarnitine (C5:1−DC)) to 18.88% (N-acetylalanine) in serum metabolites, 5.94% in urine alpha-ketoglutorate, and 4.14% in stool 5-methylhexanoate (i7:0). CKD stage G1-G2 did not affect saliva metabolites. A step-by-step report of the mediation models used is reported in Tables S2–S5.

The major super pathways involved with metabolites that mediated the association between DAL→CKD stage G1-G2 were mainly amino acid (n = 6) and lipid metabolism (n = 2) (Table S7) across all biological samples. Spearman’s correlation showed that the metabolites strongly correlate with each other, even across biological fluids (Figure 3D).

### DAL and the gut microbiota

**Figure 4:**
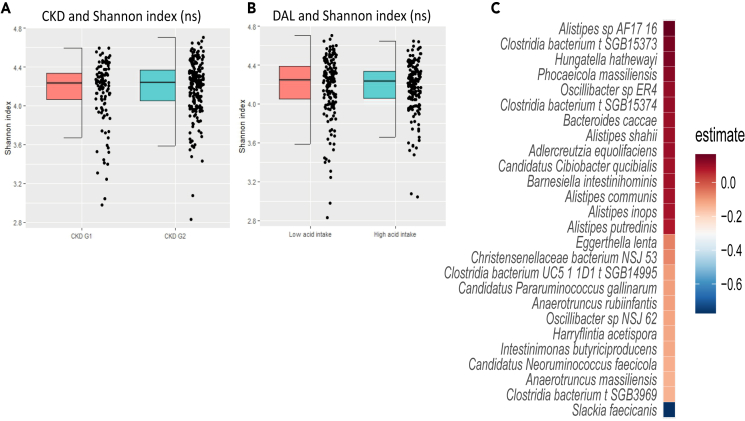
Gut microbiota, CKD and DAL Effect of (A) CKD and (B) DAL on Shannon alpha diversity (ns = non-significant) and (C) gut microbiota species identified to significantly (p < 0.05; FDR corrected [Benjamini & Hochberg]) associate with the stool metabolite 5-methylhexanoate (i7:0) in the discovery cohort. All analyses are corrected for age, sex, and BMI.

Next, we explored if the metabolites mediating the DAL-CKD relation were associated with gut microbiota composition (alpha diversity, represented as Shannon index) and microbial species (Figure 4). We found that the gut microbiota could predict 13% of the variance in stool abundances of the metabolite, 5-methylhexanoate (i7:0), and we detected 26 bacterial species that were significantly associated with it. Out of these 3 were belonging to different *Clostridia bacterium* species genomic bins (SGBs).

Furthermore, we found no association between the mediating serum and urine metabolites and gut microbiota composition.

## Discussion

In this large cross-sectional study integrating serum, urine, stool, and saliva metabolites, we report, for the first time, that the effect of early-stage kidney function declines (CKD stage G1-G2) due to DAL being possibly mediated by serum, urine, and stool, but not by saliva metabolites. The metabolites mediating the association are predominantly related to amino acid (n = 6) and lipid (n = 2) metabolism and strongly correlate with each other, even across biological sample types. Moreover, RF machine learning models identified that 13% of the variance of the stool metabolite, 5-methylhexanoate (i7:0) that potentially mediates the DAL-CKD association, could be explained via the gut microbiota composition. However, it is important to note that longitudinal, as well as interventional studies, are warranted to further confirm the potential mediating effects of the found metabolites.

This study also revealed multiple microbial species linked with the mediating stool metabolite, thereby providing potential for novel treatment options.

Our finding that DAL contributes to CKD progression supports previous reports and meta-analysis.^14^^,^^15^ The underlying pathophysiology of this relationship is complex and poorly understood, with the metabolome likely to play an important role.^11^

To date, only one study has investigated the role of the serum metabolome in the DAL-CKD relationship.^13^ In this study performed by Tariq et al. (2022), the authors identified 12 metabolites to be inversely associated with DAL, of which only one (N-methylproline) was also inversely associated with incident CKD. Our study identified, through machine learning models, 3 serum metabolites to be inversely associated with both DAL and CKD, and 8 positively associated. Our study confirms that indolepropionate and tartronate (hydroxymalonate) are negatively associated with DAL, as previously reported,^16^ highlighting the robustness of our result. However, we also found these metabolites to be negatively associated with CKD. The contrast in our findings likely originates from the fact that Tariq et al. (2022) studied these associations using a population with overt CKD (stage 2–4) that also used medications known to affect the serum metabolome, such as antihypertensive drugs,^17^ as well as different metabolomic platforms to those used in our study.

Moreover, our study identified 8 serum, 2 urine, and 1 stool metabolite to potentially mediate the positive association between DAL and CKD stage G1-G2. The majority of the mediating metabolites identified play a role in amino acid metabolism, which is to be expected as DAL is largely driven by protein intake.^4^^,^^18^ Interestingly, one metabolite, 3-MH, was identified as a mediating metabolite in both serum and urine. 3-MH is a product of histidine metabolism and produced after actin and myosin degradation.^19^ 3-MH has been proposed as a marker for protein turnover but is influenced by meat intake.^20^ 3-MH, therefore, has gained attention as a metabolite that can potentially serve as a positive biomarker for meat intake, especially poultry.^21^^,^^22^ However, the effects of 3-MH on kidney function remain inconclusive. One study using rat models showed that serum 3-MH is a candidate biomarker for acute renal injury, as its levels increased when drug-induced nephrotoxicity was induced.^23^ Another human observational study showed higher levels of 3-MH in subjects with moderate kidney failure compared to healthy individuals.^24^ However, another study in individuals on hemodialysis identified low levels of 3-MH to be predictive of cardiovascular events.^25^

This study also identified the stool metabolite 5-methylhexanoate (i7:0) to potentially mediate the effects of DAL on CKD and identified several gut microbiota species that correlate with this metabolite. 5-methylhexanoate belongs to the group of medium-chain fatty acids (MCFAs) but is not well investigated as an individual metabolite. However, the effect of MCFA in general on human health has been extensively reviewed elsewhere,^26^ and was generally found to be positive for human health, but deleterious effects have also been reported.^27^ DAL itself did not associate with gut microbiota composition, which aligns with previous studies reporting that protein intake, a major source of DAL, does not affect gut microbiota diversity.^28^^,^^29^

As kidney function can also affect host metabolome,^11^^,^^30^ we also performed mediation analyses by using CKD stage as potential mediator, i.e., DAL→ CKD stage G1-G2 (potential mediator)→ metabolite of interest. Though we found that early-stage CKD partially mediates the DAL→ metabolome relationship in selected metabolites, the VAF was lower when using metabolites as the mediator. This could be explained by the fact that we only used early-stage CKD and the association may be stronger as kidney function declines toward end-stage CKD.^30^

Multiple studies have reported an intricate relationship between the gut microbiota and kidney disease,^31^^,^^32^^,^^33^^,^^34^ with the gut microbiota correlating with end-stage CKD^34^ In our data, we did not find an association between gut microbiota composition and CKD; however, our study participants were early-stage CKD (stage G1-G2).^34^ We did however find 26 microbial species to be associated with fecal abundances of the stool metabolite 5-methylhexanoate (i7:0) that mediated the association between DAL and CKD, thus linking the gut microbiome’s involvement in CKD.

Of the 26 microbial species, *Alistipes* spp. had the strongest positive association and has been consistently reported to be increased in CKD, and to be associated with an increased intake of a Western diet, high in animal protein and fat but low in fiber.^2^^,^^35^ Our study also identified *Intestimonas butyriciproducens*, a known producer of the beneficial metabolite butyrate, a short-chain fatty acid (SCFA),^36^ to be negatively associated with 5-methylhexanoate (i7:0). This finding further emphasizes a role for SCFA in line with a previous study reporting that supplementation with the probiotic *L. casei Zhang* increased SCFA levels and reduced CKD progression in mice.^31^

In conclusion, we identified serum, stool, and urinary metabolites to possibly mediate the positive relationship between DAL and CKD stage G1-G2. Moreover, this study identified several gut microbiota species, such as *Alistipes* spp and *Intestimonas butyriciproducens*, that can be used as novel targets to mitigate this relationship, suggesting that the gut microbiota may be a therapeutic option to combat the effects of DAL on kidney function decline by altering host metabolome. Longitudinal, as well as interventional, studies are warranted to investigate the exact relationship of the identified metabolites and microbial species and to elucidate potential causality in the relationship between DAL and kidney function decline.

### Limitations of the study

This study has limitations and strengths. Strengths of this study include the use of a large population-based cohort with accurate phenotyping, dietary information, and concurrent metabolomics in multiple biological fluids. Another strength of our study is that we explored the host metabolome, using the Metabolon, Inc, platform, that covers a wide range of metabolites from multiple biological pathways leading to a more detailed understanding of the underlying biology.

However, our findings should also be appreciated in the context of some limitations. First, DAL was determined using self-reported dietary intake. Although a previously validated formula was used to calculate DAL,^4^^,^^37^ this approach is prone to several biases, including misreporting bias, which may cause misclassification bias. However, there is no gold standard to measure DAL and each method has its own drawbacks.^4^

Second, our study sample was limited to individuals with normal-to-mild renal impairment and was predominantly females of Caucasian ancestry. Accordingly, our results cannot be generalized to those with moderate-to-end-stage renal disease, and future research should explore the relationships identified here in those with later-stage CKD.

Third, the mediation analysis has some limitations. Although our study identified multiple metabolites that potentially mediate the relationship between DAL and CKD progression, our data do not permit comments on causality due to the lack of a temporal relationship. Moreover, our study cannot rule out a different directionality between exposure, mediator, and outcomes. Hence, we tested both directions. These analyses indicate a potential mediating effect of CKD stage on the metabolite of interest and, to a larger extent, of the metabolite of interest on CKD.

Finally, we were unable to replicate our results in an independent sample as, to our knowledge, there are currently no cohorts with such a comprehensive assessment of serum, urine, stool, and saliva metabolomics as well as clinical creatinine, dietary data, and shotgun metagenome profiling of the gut microbiome.

Longitudinal metabolomics data, as well as dietary and kidney function, are needed to confirm causality and understand directionality.

## STAR★Methods

### Key resources table

**Table:** 

REAGENT or RESOURCE	SOURCE	IDENTIFIER
**Biological samples**		

Human plasma/urine/fecal/saliva metabolomics data	TwinsUK; measured via Metabolon,Inc, Durham, North Carolina, USA	N/A
Human fecal metagenomics data	TwinsUK	EBI (https://www.ebi.ac.uk/) accession number PRJEB32731

**Critical commercial assays**		

DNA extraction kit	QIAamp DNA Mini kit	N/A

**Deposited data**		

Dietary data	TwinsUK	twinsuk.ac.uk/resources-for-researchers/access-our-data
Anthropometric data	TwinsUK	twinsuk.ac.uk/resources-for-researchers/access-our-data

**Software and algorithms**		

open software program R version 4.2.1	( R Core Team (2022). R: A Language and Environment for Statistical Computing. R Foundation for Statistical Computing, Vienna, Austria. URL https://Www.R-Project.Org/., n.d.)	https://www.R-project.org/
FactoMineR package in R (version 2.7)	Comprehensive R Archive Network	http://factominer.free.fr/
Vegan package in R (version 2.6-4)	Comprehensive R Archive Network	https://github.com/vegandevs/vegan
randomForest R package (version 4.7.1.1)	Comprehensive R Archive Network	cran.r-project.org/web/packages/randomForest/randomForest.pdf

### Resource availability

#### Lead contact

Further information should be directed to and will be fulfilled by the lead contact Dr. Cristina Menni (cristina.menni@kcl.ac.uk).

#### Materials availability

This study did not generate new unique reagents or materials.

#### Data and code availability

• The data used in this study are held by the Department of Twin Research at King’s College London. The data can be released to bona fide researchers using our normal procedures overseen by the Wellcome Trust and its guidelines as part of our core funding (https://twinsuk.ac.uk/resources-for-researchers/access-our-data/). The gut microbiome data is available on EBI (https://www.ebi.ac.uk/) under accession PRJEB32731 (TwinsUK).
• This paper does not report original code.
• Any additional information required to reanalyze the data reported in this paper is available from the lead contact upon reasonable request.

### Experimental model and study participant details

#### Study population and ethics approval

We included 1,453 individuals from TwinsUK^38^ with good estimated kidney function (CKD stage G1), or moderately impaired (CKD stage G2) as defined by the 2021 CKD-EPI formula (based on serum creatinine).^39^ Included participants also had data on dietary intake, and metabolites measured from multiple biological samples (serum, urine, stool and saliva). TwinsUK is the largest adult twin registry in the world, recruited as research volunteers, without selecting for any particular disease or trait, and has been shown to be comparable to the general British population for lifestyle characteristics.^40^ The baseline characteristics of participants are described in Table 1.

This study was carried out under TwinsUK BioBank ethics, approved by North West – Liverpool Central Research Ethics Committee (REC reference 19/NW/0187), IRAS ID 258513. This approval supersedes earlier approvals granted to TwinsUK by the St Thomas’ Hospital Research Ethics Committee, later London – Westminster Research Ethics Committee (REC reference EC04/015), which have now been subsumed within the TwinsUK BioBank.

### Method details

#### Study design

A flowchart of the study design is presented in Figure 1. Our primary objective is to investigate the relationship between DAL and kidney function decline (CKD stage G1-G2) and to understand the metabolites underlying the relationship. Our secondary aims were (i) to explore the mediating effects of the identified metabolites per biological sample; and (ii) to identify underlying gut microbial species that drive the mediating metabolites per biological sample.

#### Metabolite profiling

Metabolite concentrations were measured at fasting from serum, urine, stool and saliva samples by Metabolon Inc. (Durham, USA) using an untargeted Liquid chromatography–mass spectrometry (LC-MS) platform as previously described.^41^ This platform was used for both the discovery, and the replication cohort. Metabolites with more than 20% missingness were excluded, and the remaining metabolites were day median normalized, imputed to the day minimum, and inverse normalized.^42^ The Metabolon platform measured 585 serum, 656 urine, 492 stool and 400 saliva metabolites post-quality check from different metabolic pathways including amino acid, lipid, carbohydrate and vitamin metabolism.

#### Metabolite measurement and standardization

To quantify metabolites, the area-under-the-curve method was employed. Each metabolite’s raw area count in every sample underwent standardization to mitigate variations caused by differences in daily instrument tuning. This was achieved by adjusting the median values for each day of the run to 1.0. Such normalization allowed for maintaining sample-to-sample variations while enabling the comparison of metabolites with significantly differing raw peak areas on a uniform scale.

#### Quality control

In addressing batch effects in metabolite measurements, the experimental sample values for each metabolite were normalized against the median value of those in the same batch. This process standardized each batch, setting the median for each metabolite at one. For missing values, imputation was done using the lowest value from all the batches in the median-adjusted data.

#### Dietary intake

Dietary intake was estimated using a modified European Prospective Investigation into Cancer and Nutrition food frequency questionnaire (FFQ).^43^ FFQs were excluded if more than 10 food items were left unanswered or if the total energy intake estimate derived from FFQ as a ratio of the subject’s estimated basal metabolic rate (determined by the Harris–Benedict equation)^44^ was more than 2 standard deviations outside the mean of this ratio (<0.52 or >2.58), as previously described.^45^

DAL was calculated as the net endogenous acid production from FFQ data using a validated formula^4^^,^^37^: Net Endogenous Acid Production (mEq H+/day) = 54.5 × [protein intake (g/day)/potassium intake (mEq/day)) − 10.2.

#### Gut microbiota fecal collection, DNA extraction and metagenome profiling

Fecal sample collection, DNA extraction and metagenome profiling were performed as previously described.^46^ Briefly, participants collected stool samples at home in pre-labelled kits (containing 2 x 25ml tube or 1 x 25ml tube and 1 x 10ml Zymo buffer) posted to them before their clinic visit date and brought with them to the visit. Alternatively, samples could be posted to the clinic using blue Royal Mail safe boxes, ensuring correct cooled temperature. In the laboratory, samples were homogenised, aliquoted into 4 bijou tubes, and stored at −80°C, within 2 hours of receipt. TwinsUK sequenced metagenomes were processed using the YAMP pipeline (v. 0.9.5.3)31.^46^ The metagenomic analysis was conducted following the general guidelines^47^ and based on the bioBakery computational environment.^48^ High-resolution taxonomic profiling of the metagenomes was performed using MetaPhlAn 4.beta.2 with the Jan21 database that comprises 26,970 species-level genome bins, with default parameters.^49^

### Quantification and statistical analysis

Statistical analyses were performed using open software program R (version 4.2.1).^50^

DAL was adjusted for energy intake using the residual method.^51^ DAL was then converted to tertiles and the top and bottom tertiles were used for analyses.

Linear and logistic mixed-effect models were used to investigate high/low DAL - CKD/eGFR associations, correcting for age, sex, body mass index (BMI) and family relatedness (random effect). CKD and DAL were dichotomous, whereas eGFR was a continuous variable.

Principal component analysis and PERMANOVA were performed using the FactoMineR (version 1.34)^52^ and vegan packages (version 2.6)^50^ to explore the serum, urine, stool, and saliva metabolite profiles of individuals in CKD stage G1 and G2 in relation to their DAL.

For each biological tissue (urine, stool, saliva, serum) two random forest (RF) models were employed to identify the most influential metabolites (from multiple biological samples) affecting (1) DAL, and (2) CKD stage G1-G2. Models were adjusted for traditional risk factors (age, sex, and BMI), by calculating the residuals per metabolite, using the risk factors as independent variables. For each RF, the dataset was split (80:20), with 80% going into a training set and 20% into the test set. Hyperparameters were tuned using the adaptive resampling search, and the optimum number of features was calculated using five-fold cross-validation and selected by node purity. Using the optimum number of metabolites from the RF model, we generated a panel of metabolites (per biological sample) that overlapped between the DAL model and the CKD model.

To identify a direction of effect for the metabolite panels we ran regression models adjusting for covariates. The Benjamini-Hochberg method was used to correct for multiple testing (false discovery rate (FDR)<0.05).^53^

Metabolites that passed the multiple testing threshold (FDR; p<0.05), were tested for a potential mediating effect in the relationship between DAL and CKD stage G1-G2. Mediation analysis was performed using the ‘mediate’ function in the R package ‘mediation’ (version 4.5.0).^54^ The variance accounted for (VAF) was determined as the ratio of indirect-to-total effect and distinguishes the proportion of the variance explained by the mediation process (the proportion of the effect of DAL on CKD status that goes through the metabolite). Mediation analyses was conducted in accordance with the AGReMA guidelines for conducting and reporting mediation analyses (Table S1).^55^

The main mediation model was constructed as DAL (independent variable)→ Metabolite of interest (potential mediator) → CKD stage G1-G2 (dependent variable).

However, as kidney function is also associated with changes in metabolites^30^ we also tested the model DAL(independent variable)→ CKD stage G1-G2 (potential mediator)→ metabolite of interest (dependent variable). All available data was used for the mediation models and no sample size calculation was conducted prior to the analyses, as is often the case in mediation analyses.^55^ All models and corresponding output are reported in Tables S2–S5.

The relationship between the mediatory metabolites and the gut microbiota was then tested using multivariable regression models with FDR correction. Explained variance of the gut microbiota with mediating metabolites was determined using RF models as described above. Alpha diversity (Shannon index) was calculated using the “vegan” package (version 2.6).^56^

## Supplemental information

Document S1. Figures S1 and S2 and Tables S1–S5

Table S6. Serum, urine, stool, and saliva metabolites identified through random forest machine learning that correspond with both dietary acid load as well as CKD stage G1-G2, related to Figure 2

Table S7. Metabolites mediating the association between DAL and CKD stage G1-G2 across different biological samples, related to Figure 2
