# Supplementary material for: Cross-sectional analyses of metabolites across biological samples mediating dietary acid load and chronic kidney disease
Source: iScience. 2024 Feb 5;27(3):109132. doi: 10.1016/j.isci.2024.109132 (PMC10907771; doi:10.1016/j.isci.2024.109132)
Supplement: Document S1. Figures S1 and S2 and Tables S1–S5 [file mmc1.pdf]

iScience, Volume *27*

Supplemental information

Cross-sectional analyses of metabolites across biological samples mediating dietary acid load and chronic kidney disease

Ilias Attaye, Beverley Beynon-Cobb, Panayiotis Louca, Ana Nogal, Alessia Visconti, Francesca Tettamanzi, Kari Wong, Gregory Michellotti, Tim D. Spector, Mario Falchi, Jordana T. Bell, and Cristina Menni

Supplementary table S1: AGReMA checklist, related to figure 1

Section/Topic	Item Number	Item Description	Reported on page No
**Title and abstract**			
Title	1	Identify that the study uses mediation analysis	1
Abstract	2	Provide a structured summary of the objectives, methods, results, and conclusions specific to mediation analyses	2
**Introduction**			
Background and rationale	3	Describe the study background and theoretical rationale for investigating the mechanisms of interest. Include supporting evidence or theoretical rationale for why the intervention or exposure might have a causal relationship with the proposed mediators. Include supporting evidence or theoretical rationale for why the mediators might have a causal relationship with the outcomes	3
Objectives	4	State the objectives of the study specific to the mechanisms of interest. The objectives should specify whether the study aims to test or estimate the mechanistic effects	3
**Methods**			
Study registration	5	If applicable, provide references to any protocols or study registrations specific to the mediation analysis, and highlight any deviations from the planned protocol	NA
Study design and source of data	6	Specify the design of the original study that was used in mediation analyses and where the details can be accessed, supported by a reference. If applicable, describe study design features that are relevant to mediation analyses	4
Participants	7	Describe the target population, eligibility criteria specific to mediation analyses, study locations, and study dates (start of participant enrolment and end of follow-up)	4
Sample Size	8	State whether a sample size calculation was conducted for mediation analyses. If so, explain how it was calculated	6
Effects of interest	9	Specify the effects of interest	6
Assumed causal model	10	Include a graphic representation of the assumed causal model including the exposure, mediator, outcome, and possible confounders	4
Causal assumptions	11	Specify assumptions about the causal model	6
Measurement	12	Clearly describe the interventions or exposures, mediators, outcomes, confounders, and moderators that were used in the analyses. Specify how and when they were measured, the measurement properties, and whether blinded assessment was used	5-6
Measurement levels	13	If relevant, describe the levels at which the exposure, mediator, and outcome were measured	NA

(cc) BY-NC-ND

Statistical methods	14	Describe the statistical methods used to estimate the causal relationships of interest. This description should specify analytical strategies used to reduce confounding, model building procedures, justification for the inclusion or exclusion of possible interaction terms, modelling assumptions, and methods used to handle missing data. Provide a reference to the statistical software and package used	5-6
Sensitivity analyses	15	Describe any sensitivity analyses that were used to explore causal or statistical assumptions and the influence of missing data	NA
Ethical approval	16	Name the institutional research board or ethics committee that approved the study. Provide a description of participant informed consent or ethics committee waiver of informed consent	7
**Results**			
Participants	17	Describe baseline characteristics of participants included in mediation analyses. Report the total sample size and number of participants lost during follow-up or with missing data	7
Outcomes and estimates	18	Report point estimates and uncertainty estimates for the exposure-mediator and mediator-outcome relationships. If inference concerning the causal relationship of interest is considered feasible given the causal assumptions, report the point estimate and uncertainty estimate	8
Sensitivity parameters	19	Report the results from any sensitivity analyses used to assess robustness of the causal or statistical assumptions, and the influence of missing data	NA
**Discussion**			
Limitations	20	Discuss the limitations of the study including potential sources of bias	12
Interpretation	21	Interpret the estimated effects considering the study's magnitude and uncertainty, plausibility of the causal assumptions, limitations, generalizability of the findings, and results from relevant studies	10-12
Implications	22	Discuss the implications of the overall results for clinical practice, policy, and science	10-12
**Other information**			
Funding and role of sponsor	23	List all sources of funding or sponsorship for the mediation analysis and the role of the funders/sponsors in the conduct of the study, writing of the manuscript, and decision to submit for publication.	13
Conflicts of interest and financial disclosures	24	State any conflicts of interest and financial disclosures for all authors	12
Data and code	25	Authors are encouraged to provide a statement for sharing data and code for the mediation analysis	14

From: Lee H, Cashin AG, Lamb SE, Hopewell S, Vansteelandt S, VanderWeele TJ, et al. A Guideline for Reporting Mediation Analyses of Randomized Trials and Observational Studies. The AGReMA Statement. JAMA. 2021;326(11):1045–1056. doi:10.1001/jama.2021.14075

AGReMA is designed for articles that report mediation analyses of randomized trials or observational studies.
For more information, visit: agrema-statement.org

(cc) BY-NC-ND

Table S2: results of regression models used for mediation analyses with serum metabolites Related to figure 2.

Model 0=CKD~ acid intake+age+sex+bmi
Model 1=metabolite~ acid intake + age+ sex+ bmi
Model 2 CKD~ acid intake+metabolite+age+sex+bmi
Model 3 metabolite~ acid intake+age+sex+bmi
Model 4 CKD~ acid intake + age+ sex+ bmi
Model 5 metabolite~ acid intake+CKD+age+sex+bmi
VAF-1= Variance Accounted For using metabolite as mediator; VAF-2= Variance Accounted For using CKD as mediator;
ACME-1=Average Causal Mediation Effects using metabolite as mediator; AMCE-2= Average Causal Mediation Effects using CKD as mediator

**Model 0_1–methyl–5–imidazoleacetate**				**Model 1_1–methyl–5–imidazoleacetate**				**Model 2_1–methyl–5–imidazoleacetate**			
	**estimate**	**SE**	**p-value**		**estimate**	**SE**	**p-value**		**estimate**	**SE**	**p-value**
acid intake	0.32	0.15	0.03	acid intake	0.22	0.06	4.00E-04	acid intake	0.23	0.15	0.13
								metabolite	0.53	0.08	4.66E-11
ACME-1=	β=0.022; p<2E-16			VAF-1=26.72%							
**Model 3_1–methyl–5–imidazoleacetate**				**Model 4_1–methyl–5–imidazoleacetate**				**Model 5_1–methyl–5–imidazoleacetate**			
	**estimate**	**SE**	**p-value**		**estimate**	**SE**	**p-value**		**estimate**	**SE**	**p-value**
acid intake	0.22	0.06	4.00E-04	acid intake	0.06	0.03	0.03	acid intake	0.19	0.06	1.73E-03
								CKD	0.46	0.07	1.09E-11
ACME-2=	β=0.030; p=0.042			VAF-2=11.15%							

**Model 0_3,5–dichloro–2,6–dihydroxybenzoic acid**				**Model 1_3,5–dichloro–2,6–dihydroxybenzoic acid**				**Model 2_3,5–dichloro–2,6–dihydroxybenzoic acid**			
	**estimate**	**SE**	**p-value**		**estimate**	**SE**	**p-value**		**estimate**	**SE**	**p-value**
acid intake	0.32	0.15	0.03	acid intake	0.28	0.06	4.46E-06	acid intake	0.28	0.15	0.06
								metabolite	0.16	0.08	0.03
ACME-1=	β=0.009; p=0.040			VAF-1=12.28%							

Model 3_3,5−dichloro−2,6−dihydroxybenzoic acid				Model 4_3,5−dichloro−2,6−dihydroxybenzoic acid				Model 5_3,5−dichloro−2,6−dihydroxybenzoic acid			
	estimate	SE	p-value		estimate	SE	p-value		estimate	SE	p-value
acid intake	0.28	0.06	4.46E-06	acid intake	0.06	0.03	0.03	acid intake	0.27	0.06	9.79E-06
								CKD	0.16	0.07	0.02
ACME-2=	β= 1.03E-02; p=0.054			VAF-2=NA; no significant mediation							

Model 0_3−methylhistidine				Model 1_3−methylhistidine				Model 2_3−methylhistidine			
	estimate	SE	p-value		estimate	SE	p-value		estimate	SE	p-value
acid intake	0.32	0.15	0.03	acid intake	0.23	0.06	0.00	acid intake	0.25	0.15	0.09
								metabolite	0.39	0.08	0.00
ACME-1=	β=0.017; p<2E-16			VAF-1=21.89%							

Model 3_3−methylhistidine				Model 4_3−methylhistidine				Model 5_3−methylhistidine			
	estimate	SE	p-value		estimate	SE	p-value		estimate	SE	p-value
acid intake	0.25	0.06	5.20E-05	acid intake	0.06	0.03	0.03	acid intake	0.24	0.06	9.99E-05
								CKD	0.15	0.07	0.03
ACME-2=	β=0.021; p=0.030			VAF-2=3.47%							

Model 0_deoxycarnitine				Model 1_deoxycarnitine				Model 2_deoxycarnitine			
	estimate	SE	p-value		estimate	SE	p-value		estimate	SE	p-value
acid intake	0.32	0.15	0.03	acid intake	0.24	0.06	9.57E-05	acid intake	0.15	0.15	0.32
								metabolite	0.78	0.09	5.91E-19
ACME-1=	β=0.034; p<2E-16			VAF-1=36.91%							

	Model 3_deoxycarnitine			Model 4_deoxycarnitine				Model 5_deoxycarnitine		
	estimate	SE	p-value		estimate	SE	p-value		estimate	SE	p-value
acid intake	0.24	0.06	9.57E-05	acid intake	0.06	0.03	0.03	acid intake	0.20	0.06	6.85E-04
								CKD	0.63	0.06	2.86E-21
ACME-2=	β=0.040; p=0.024			VAF-2=13.61%							

	Model 0_N1-methylinosine				Model 1_N1-methylinosine				Model 2_N1-methylinosine		
	estimate	SE	p-value		estimate	SE	p-value		estimate	SE	p-value
acid intake	0.32	0.15	0.03	acid intake	0.19	0.06	8.83E-04	acid intake	0.18	0.15	0.24
								metabolite	0.83	0.09	2.46E-19
ACME-1=	β=0.029; p=0.002			VAF-1=33.03%							

	Model 3_N1-methylinosine				Model 4_N1-methylinosine				Model 5_N1-methylinosine		
	estimate	SE	p-value		estimate	SE	p-value		estimate	SE	p-value
acid intake	0.19	0.06	8.83E-04	acid intake	0.06	0.03	0.03	acid intake	0.15	0.06	0.01
								CKD	0.60	0.06	3.16E-22
ACME-2=	β=0.039; p=0.026			VAF-2=15.93%							

	Model 0_N6,N6,N6-trimethyllysine				Model 1_N6,N6,N6-trimethyllysine				Model 2_N6,N6,N6-trimethyllysine		
	estimate	SE	p-value		estimate	SE	p-value		estimate	SE	p-value
acid intake	0.32	0.15	0.03	acid intake	0.20	0.07	2.30E-03	acid intake	0.22	0.15	0.15
								metabolite	0.58	0.08	2.75E-14
ACME-1=	β=0.022; p=0.002			VAF-1=26.61%							

Model 3_N6,N6,N6-trimethyllysine	Model 4_N6,N6,N6-trimethyllysine	Model 5_N6,N6,N6-trimethyllysine

	estimate	SE	p-value		estimate	SE	p-value		estimate	SE	p-value
acid intake	0.20	0.07	2.30E-03	acid intake	0.06	0.03	0.03	acid intake	0.17	0.06	0.01
								CKD	0.58	0.07	1.29E-15
ACME-2=	β=0.037; p=0.030			VAF-2=14.82%							

Model 0_N-acetylalanine				Model 1_N-acetylalanine				Model 2_N-acetylalanine			
	estimate	SE	p-value		estimate	SE	p-value		estimate	SE	p-value
acid intake	0.32	0.15	0.03	acid intake	0.17	0.05	2.03E-03	acid intake	0.18	0.16	0.25
								metabolite	1.09	0.11	4.48E-25
ACME-1=	β=0.032; p=0.002			VAF-1=36.65%							

Model 3_N-acetylalanine				Model 4_N-acetylalanine				Model 5_N-acetylalanine			
	estimate	SE	p-value		estimate	SE	p-value		estimate	SE	p-value
acid intake	0.17	0.05	2.03E-03	acid intake	0.06	0.03	0.03	acid intake	0.12	0.05	0.01
								CKD	0.66	0.06	1.89E-30
ACME-2=	β=0.042; p=0.030			VAF-2=18.88%							

Model 0_tiglylcarnitine (C5:1−DC)				Model 1_tiglylcarnitine (C5:1−DC)				Model 2_tiglylcarnitine (C5:1−DC)			
	estimate	SE	p-value		estimate	SE	p-value		estimate	SE	p-value
acid intake	0.32	0.15	0.03	acid intake	0.21	0.06	6.89E-04	acid intake	0.26	0.15	0.08
								metabolite	0.35	0.08	3.97E-06
ACME-1=	β=0.015; p=0.002			VAF-1=18.69%							

Model 3_tiglylcarnitine (C5:1−DC)				Model 4_tiglylcarnitine (C5:1−DC)				Model 5_tiglylcarnitine (C5:1−DC)			
	estimate	SE	p-value		estimate	SE	p-value		estimate	SE	p-value

acid intake	0.21	0.06	6.89E-04	acid intake	0.06	0.03	0.03	acid intake	0.19	0.06	1.97E-03
								CKD	0.32	0.07	3.80E-06
ACME-2=	β=0.020; p=0.030			VAF-2=8.38%							

Table S3: results of regression models used for mediation analyses with urine metabolites. Related to figure 2

Model 0=CKD~ acid intake+age+sex+bmi
Model 1=metabolite~ acid intake + age+ sex+ bmi
Model 2 CKD~ acid intake+metabolite+age+sex+bmi
Model 3 metabolite~ acid intake+age+sex+bmi
Model 4 CKD~ acid intake + age+ sex+ bmi
Model 5 metabolite~ acid intake+CKD+age+sex+bmi
VAF-1= Variance Accounted For using metabolite as mediator; VAF-2= Variance Accounted For using CKD as mediator;
ACME-1=Average Causal Mediation Effects using metabolite as mediator; AMCE-2= Average Causal Mediation Effects using CKD as mediator

**Model 0_3−hydroxyphenylacetate**				**Model 1_3−hydroxyphenylacetate**				**Model 2_3−hydroxyphenylacetate**			
	**estimate**	**SE**	**p-value**		**estimate**	**SE**	**p-value**		**estimate**	**SE**	**p-value**
acid intake	0.32	0.15	0.03	acid intake	0.05	0.06	0.39	acid intake	0.33	0.15	0.02
								metabolite	-0.06	0.07	0.38
ACME-1=	β= -0.0007 ; p=0.616			VAF-1=NA; no significant mediation							
**Model 3_3−hydroxyphenylacetate**				**Model 4_3−hydroxyphenylacetate**				**Model 5_3−hydroxyphenylacetate**			
	**estimate**	**SE**	**p-value**		**estimate**	**SE**	**p-value**		**estimate**	**SE**	**p-value**
acid intake	0.05	0.06	0.39	acid intake	0.06	0.03	0.03	acid intake	0.06	0.03	0.03
								CKD	-0.06	0.07	0.42
ACME-2=	β=-0.004; p=0.40			VAF-2=NA; no significant mediation							

**Model 0_3−methylcrotonylglycine**				**Model 1_3−methylcrotonylglycine**				**Model 2_3−methylcrotonylglycine**			
	**estimate**	**SE**	**p-value**		**estimate**	**SE**	**p-value**		**estimate**	**SE**	**p-value**
acid intake	0.32	0.15	0.03	acid intake	-0.01	0.06	0.90	acid intake	0.32	0.15	0.03
								metabolite	0.12	0.07	0.10
ACME-1=	β= -0.0002; p=0.90			VAF-1=NA; no significant mediation							

Model 3_3−methylcrotonylglycine				Model 4_3−methylcrotonylglycine				Model 5_3−methylcrotonylglycine			
	**estimate**	**SE**	**p-value**		**estimate**	**SE**	**p-value**		**estimate**	**SE**	**p-value**
acid intake	-0.01	0.06	0.90	acid intake	0.06	0.03	0.03	acid intake	-0.01	0.06	0.90
								CKD	0.12	0.07	0.09
ACME-2=	β=0.008 ; p=0.13			VAF-2=NA; no significant mediation							

Model 0_3−methylglutarylcarnitine (2)				Model 1_3−methylglutarylcarnitine (2)				Model 2_3−methylglutarylcarnitine (2)			
	**estimate**	**SE**	**p-value**		**estimate**	**SE**	**p-value**		**estimate**	**SE**	**p-value**
acid intake	0.32	0.15	0.03	acid intake	0.12	0.06	0.05	acid intake	0.31	0.15	0.04
								metabolite	0.13	0.08	0.09
ACME-1=	β=0.003; p=0.11			VAF-1=NA; no significant mediation							
**Model 3_3−methylglutarylcarnitine (2)**				**Model 4_3−methylglutarylcarnitine (2)**				**Model 5_3−methylglutarylcarnitine (2)**			
	**estimate**	**SE**	**p-value**		**estimate**	**SE**	**p-value**		**estimate**	**SE**	**p-value**
acid intake	0.12	0.06	0.05	acid intake	0.06	0.03	0.03	acid intake	0.11	0.06	0.07
								CKD	0.11	0.07	0.09
ACME-2=	β=0.007; p= 0.098			VAF-2=NA; no significant mediation							

Model 0_3−methylhistidine				Model 1_3−methylhistidine				Model 2_3−methylhistidine			
	**estimate**	**SE**	**p-value**		**estimate**	**SE**	**p-value**		**estimate**	**SE**	**p-value**
acid intake	0.32	0.15	0.03	acid intake	0.25	0.06	0.00	acid intake	0.28	0.15	0.05
								metabolite	0.16	0.08	0.03
ACME-1=	β=0.008; p=0.040			VAF-1=11.11%							
**Model 3_3−methylhistidine**				**Model 4_3−methylhistidine**				**Model 5_3−methylhistidine**			
	**estimate**	**SE**	**p-value**		**estimate**	**SE**	**p-value**		**estimate**	**SE**	**p-value**
acid intake	0.25	0.06	0.00	acid intake	0.06	0.03	0.03	acid intake	0.24	0.06	0.00
								CKD	0.15	0.07	0.03
ACME-2=	β=0.0096; p=0.072			VAF-2=NA; no significant mediation							

Model 0_4−ethylphenol glucuronide				Model 1_4−ethylphenol glucuronide				Model 2_4−ethylphenol glucuronide			
	**estimate**	**SE**	**p-value**		**estimate**	**SE**	**p-value**		**estimate**	**SE**	**p-value**
acid intake	0.32	0.15	0.03	acid intake	0.05	0.06	0.42	acid intake	0.32	0.15	0.03
								metabolite	-0.02	0.08	0.76
ACME-1=	β= -0.0002; p=0.846			VAF-1=NA; no significant mediation							
**Model 3_4−ethylphenol glucuronide**				**Model 4_4−ethylphenol glucuronide**				**Model 5_34−ethylphenol glucuronide**			
	**estimate**	**SE**	**p-value**		**estimate**	**SE**	**p-value**		**estimate**	**SE**	**p-value**
acid intake	0.05	0.06	0.42	acid intake	0.06	0.03	0.03	acid intake	0.05	0.06	0.41
								CKD	-0.02	0.07	0.72
ACME-2=	β=-0.00153 ; p=0.73			VAF-2=NA; no significant mediation							

Model 0_alpha−ketoglutarate				Model 1_alpha−ketoglutarate				Model 2_alpha−ketoglutarate			
	**estimate**	**SE**	**p-value**		**estimate**	**SE**	**p-value**		**estimate**	**SE**	**p-value**
acid intake	0.32	0.15	0.03	acid intake	0.19	0.06	0.00	acid intake	0.29	0.15	0.05
								metabolite	0.21	0.07	0.01
ACME-1=	β= 0.00772; p=0.006			VAF-1=11.09%							
**Model 3_alpha−ketoglutarate**				**Model 4_alpha−ketoglutarate**				**Model 5_alpha−ketoglutarate**			
	**estimate**	**SE**	**p-value**		**estimate**	**SE**	**p-value**		**estimate**	**SE**	**p-value**
acid intake	0.19	0.06	0.00	acid intake	0.06	0.03	0.03	acid intake	0.18	0.06	0.01
								CKD	0.20	0.07	0.00
ACME-2=	β=0.012712;p=0.036			VAF-2=5.94%							

Model 0_cortisol 21−sulfate				Model 1_cortisol 21−sulfate				Model 2_cortisol 21−sulfate			
	**estimate**	**SE**	**p-value**		**estimate**	**SE**	**p-value**		**estimate**	**SE**	**p-value**
acid intake	0.32	0.15	0.03	acid intake	0.12	0.06	0.05	acid intake	0.33	0.15	0.02
								metabolite	-0.09	0.08	0.27
ACME-1=	β=-0.002 ; p=0.342			VAF-1=NA; no significant mediation							

Model 3_cortisol 21−sulfate				Model 4_cortisol 21−sulfate				Model 5_cortisol 21−sulfate			
	**estimate**	**SE**	**p-value**		**estimate**	**SE**	**p-value**		**estimate**	**SE**	**p-value**
acid intake	0.12	0.06	0.05	acid intake	0.06	0.03	0.03	acid intake	0.12	0.06	0.04
								CKD	-0.07	0.07	0.29
ACME-2=	β=-0.004 ; p=0.376			VAF-2=NA; no significant mediation							

Table S4: results of regression models used for mediation analyses with stool metabolites.Related to figure 2

Model 0=CKD~ acid intake+age+sex+bmi
Model 1=metabolite~ acid intake + age+ sex+ bmi
Model 2 CKD~ acid intake+metabolite+age+sex+bmi
Model 3 metabolite~ acid intake+age+sex+bmi
Model 4 CKD~ acid intake + age+ sex+ bmi
Model 5 metabolite~ acid intake+CKD+age+sex+bmi
VAF-1= Variance Accounted For using metabolite as mediator; VAF-2= Variance Accounted For using CKD as mediator;
ACME-1=Average Causal Mediation Effects using metabolite as mediator; AMCE-2= Average Causal Mediation Effects using CKD as mediator

Model 0_4–hydroxyphenylpyruvate				Model 1_4–hydroxyphenylpyruvate				Model 2_4–hydroxyphenylpyruvate			
	estimate	SE	p-value		estimate	SE	p-value		estimate	SE	p-value
acid intake	0.31	0.14	0.03	acid intake	0.00	0.06	0.95	acid intake	0.31	0.14	0.03
								metabolite	0.08	0.07	0.28
ACME-1=	β= -6.15e-05 ; p=0.904			VAF-1=NA; no significant mediation							
**Model 3_4–hydroxyphenylpyruvate**				**Model 4_4–hydroxyphenylpyruvate**				**Model 5_4–hydroxyphenylpyruvate**			
	estimate	SE	p-value		estimate	SE	p-value		estimate	SE	p-value
acid intake	0.00	0.06	0.95	acid intake	0.06	0.03	0.04	acid intake	-0.01	0.06	0.89
								CKD	0.08	0.07	0.24
ACME-2=	β=0.00498; p=0.28			VAF-2=NA; no significant mediation							

Model 0_5–methylhexanoate (i7:0)				Model 1_5–methylhexanoate (i7:0)				Model 2_5–methylhexanoate (i7:0)			
	estimate	SE	p-value		estimate	SE	p-value		estimate	SE	p-value
acid intake	0.31	0.14	0.03	acid intake	0.25	0.06	0.00	acid intake	0.26	0.15	0.07
								metabolite	0.19	0.08	0.01
ACME-1=	β= 0.0097; p=0.014			VAF-1=13.29%							

Model 3_5–methylhexanoate (i7:0)	estimate	SE	p-value	Model 4_5–methylhexanoate (i7:0)	estimate	SE	p-value	Model 5_5–methylhexanoate (i7:0)	estimate	SE	p-value
acid intake	0.25	0.06	4.51E-05	acid intake	0.06	0.03	0.04	acid intake	0.24	0.06	0.00
								CKD	0.18	0.07	0.01
ACME-2=	β=0.011; p=0.04			VAF-2=4.14%							

Model 0_pyridoxal	estimate	SE	p-value	Model 1_pyridoxal	estimate	SE	p-value	Model 2_pyridoxal	estimate	SE	p-value
acid intake	0.31	0.14	0.03	acid intake	-0.15	0.06	0.02	acid intake	0.29	0.15	0.05
								metabolite	-0.13	0.07	0.07
ACME-1=	β= 4.01e-03 ; p=0.104			VAF-1=NA; no significant mediation							

Model 3_pyridoxal	estimate	SE	p-value	Model 4_pyridoxal	estimate	SE	p-value	Model 5_pyridoxal	estimate	SE	p-value
acid intake	-0.15	0.06	0.02	acid intake	0.06	0.03	0.04	acid intake	-0.14	0.06	0.02
								CKD	-0.12	0.07	0.07
ACME-2=	β=-0.0077; p=0.124			VAF-2=NA; no significant mediation							

SupTable S5: results of regression models used for mediation analyses with saliva metabolites. Related to figure 2

Model 0=CKD~ acid intake+age+sex+bmi
Model 1=metabolite~ acid intake + age+ sex+ bmi
Model 2 CKD~ acid intake+metabolite+age+sex+bmi
Model 3 metabolite~ acid intake+age+sex+bmi
Model 4 CKD~ acid intake + age+ sex+ bmi
Model 5 metabolite~ acid intake+CKD+age+sex+bmi
VAF-1= Variance Accounted For using metabolite as mediator; VAF-2= Variance Accounted For using CKD as mediator;
ACME-1=Average Causal Mediation Effects using metabolite as mediator; AMCE-2= Average Causal Mediation Effects using CKD as mediator

Model 0_1-stearoyl-GPI (18:0)				Model 1_1-stearoyl-GPI (18:0)				Model 2_1-stearoyl-GPI (18:0)			
	**estimate**	**SE**	**p-value**		**estimate**	**SE**	**p-value**		**estimate**	**SE**	**p-value**
acid intake	0.34	0.14	0.02	acid intake	0.03	0.07	0.63	acid intake	0.34	0.14	0.02
								metabolite	-0.06	0.07	0.36
ACME-1=	β= -0.000415 ; p=0.770			VAF-1=NA; no significant mediation							
**Model 3_1-stearoyl-GPI (18:0)**				**Model 4_1-stearoyl-GPI (18:0)**				**Model 5_1-stearoyl-GPI (18:0)**			
	**estimate**	**SE**	**p-value**		**estimate**	**SE**	**p-value**		**estimate**	**SE**	**p-value**
acid intake	0.03	0.07	0.63	acid intake	0.07	0.03	0.02	acid intake	0.04	0.07	0.58
								CKD	-0.07	0.07	0.36
ACME-2=	β=-0.005; p=0.35			VAF-2=NA; no significant mediation							

Model 0_allantoin				Model 1_allantoin				Model 2_allantoin			
	**estimate**	**SE**	**p-value**		**estimate**	**SE**	**p-value**		**estimate**	**SE**	**p-value**
acid intake	0.34	0.14	0.02	acid intake	0.06	0.07	0.35	acid intake	0.33	0.14	0.02
								metabolite	0.12	0.07	0.10
ACME-1=	β= 0.00147 ; p=0.422			VAF-1=NA; no significant mediation							

	Model 3_allantoin				Model 4_allantoin				Model 5_allantoin		
	**estimate**	**SE**	**p-value**		**estimate**	**SE**	**p-value**		**estimate**	**SE**	**p-value**
acid intake	0.06	0.07	0.35	acid intake	0.07	0.03	0.02	acid intake	0.05	0.07	0.41
								CKD	0.12	0.07	0.11
ACME-2=	β=0.00796; p=0.12			VAF-2=NA; no significant mediation							
**Model 4_fructose 1,6−diphosphate/glucose 1,6−diphosphate/myo−inositol diphosphates**				**Model 4_fructose 1,6−diphosphate/glucose 1,6−diphosphate/myo−inositol diphosphates**				**Model 4_fructose 1,6−diphosphate/glucose 1,6−diphosphate/myo−inositol diphosphates**			
	**estimate**	**SE**	**p-value**		**estimate**	**SE**	**p-value**		**estimate**	**SE**	**p-value**
acid intake	0.34	0.14	0.02	acid intake	-0.05	0.06	0.43	acid intake	0.34	0.14	0.02
								metabolite	0.05	0.07	0.51
ACME-1=	β= -0.000496 ; p=0.722			VAF-1=NA; no significant mediation							
**Model 4_fructose 1,6−diphosphate/glucose 1,6−diphosphate/myo−inositol diphosphates**				**Model 4_fructose 1,6−diphosphate/glucose 1,6−diphosphate/myo−inositol diphosphates**				**Model 5_fructose 1,6−diphosphate/glucose 1,6−diphosphate/myo−inositol diphosphates**			
	**estimate**	**SE**	**p-value**		**estimate**	**SE**	**p-value**		**estimate**	**SE**	**p-value**
acid intake	-0.05	0.06	0.43	acid intake	0.07	0.03	0.02	acid intake	-0.05	0.06	0.40
								CKD	0.05	0.07	0.51
ACME-2=	β=0.00316; p=0.51			VAF-2=NA; no significant mediation							

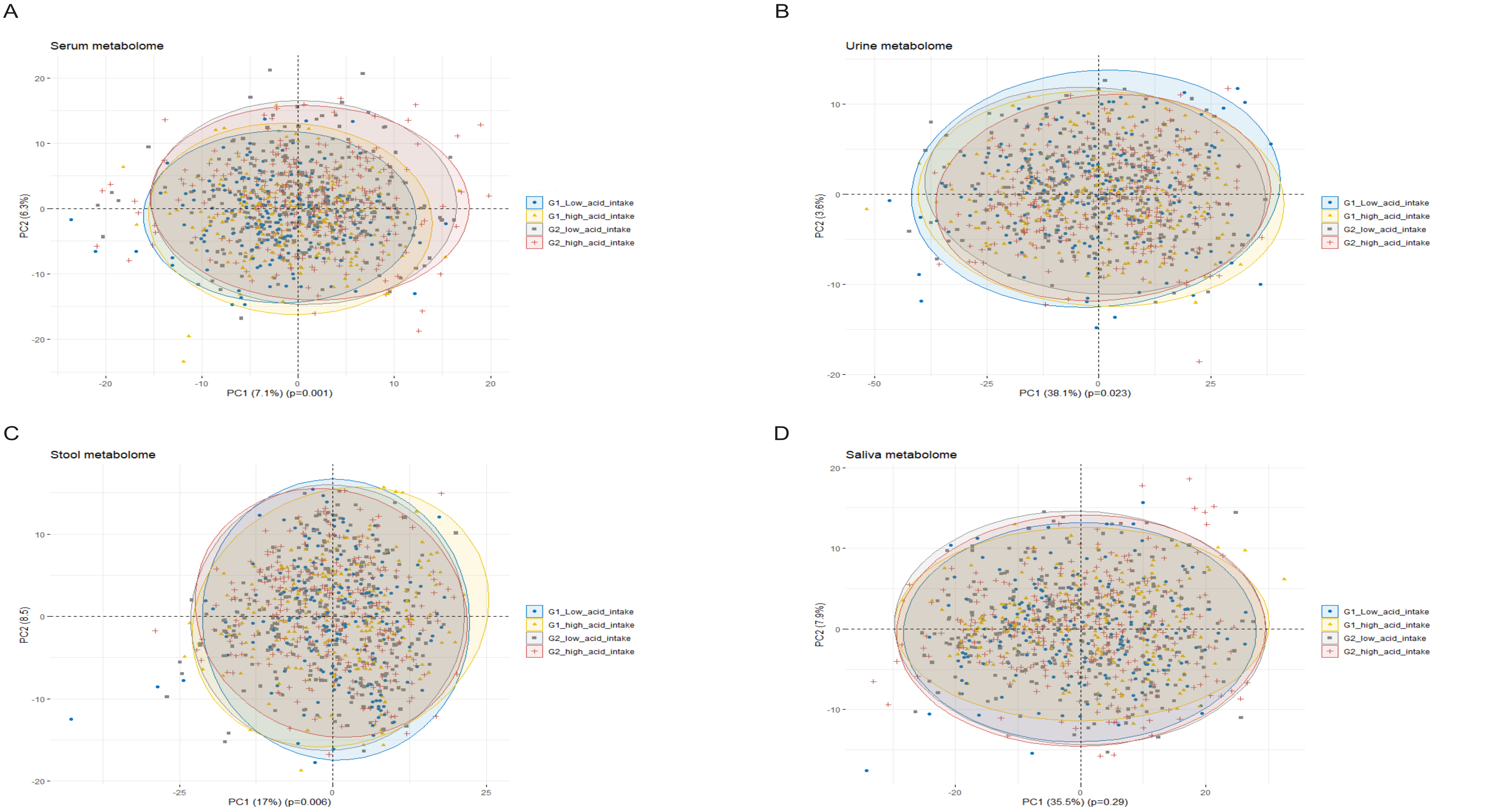

Figure S1.Results from PERMANOVA on (A) serum, (B), urine, (C) stool and (D) saliva metabolome. related to Figure 2.

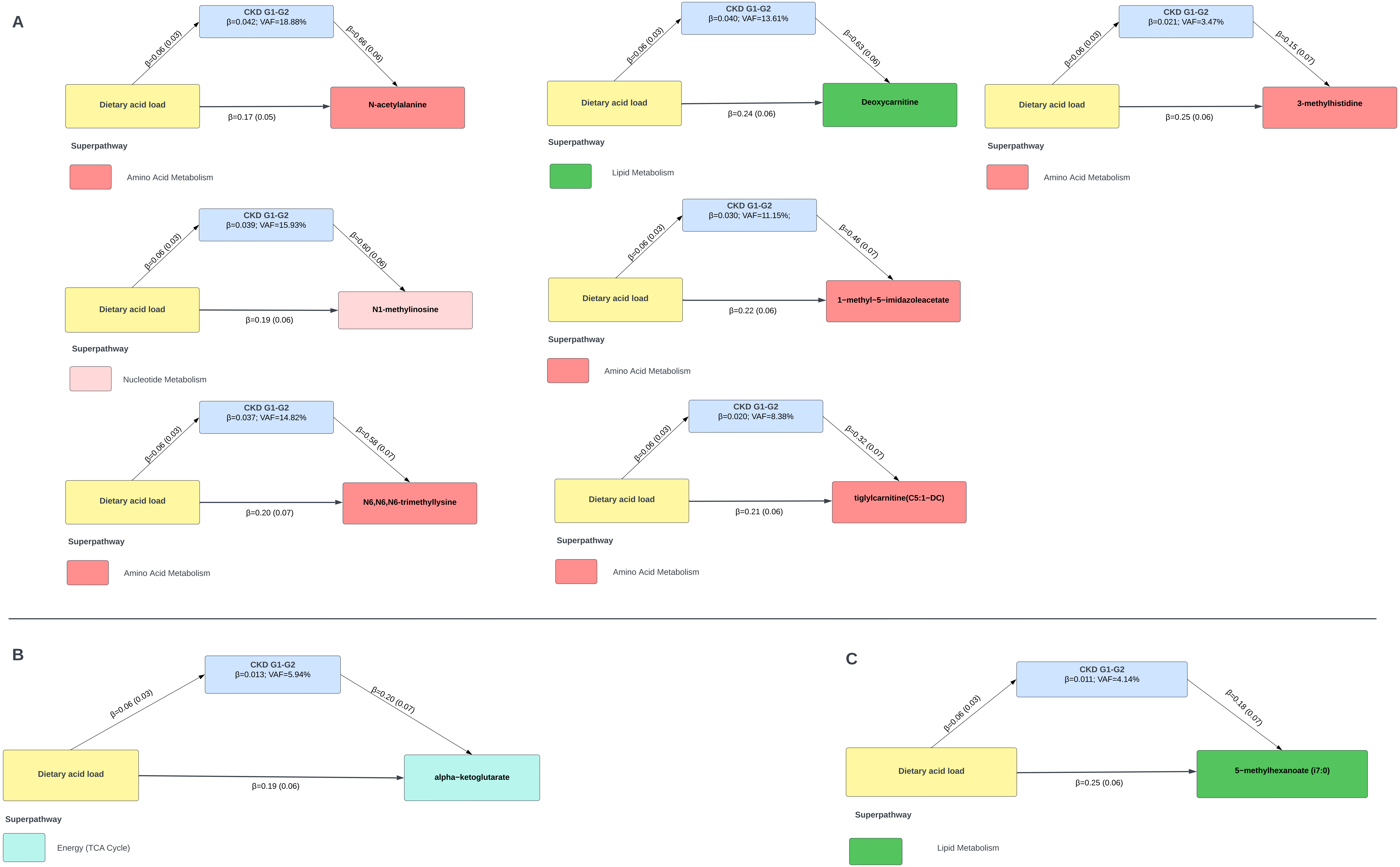

Figure S2.Mediation analysis of the association between DAL and metabolite of interest using CKD stage G1-G2 as potential mediator in (A) serum,(B) urine and (C) stool related to Figure 2.
